# High Resolution Structure of the *ba3* Cytochrome *c* Oxidase from *Thermus thermophilus* in a Lipidic Environment

**DOI:** 10.1371/journal.pone.0022348

**Published:** 2011-07-21

**Authors:** Theresa Tiefenbrunn, Wei Liu, Ying Chen, Vsevolod Katritch, C. David Stout, James A. Fee, Vadim Cherezov

**Affiliations:** 1 Department of Molecular Biology, The Scripps Research Institute, La Jolla, California, United States of America; 2 Skaggs School of Pharmacy & Pharmaceutical Sciences and San Diego Supercomputer Center, University of California, San Diego, La Jolla, California, United States of America; Institut Pasteur, France

## Abstract

The fundamental chemistry underpinning aerobic life on Earth involves reduction of dioxygen to water with concomitant proton translocation. This process is catalyzed by members of the heme-copper oxidase (HCO) superfamily. Despite the availability of crystal structures for all types of HCO, the mode of action for this enzyme is not understood at the atomic level, namely how vectorial H^+^ and e^-^ transport are coupled. Toward addressing this problem, we report wild type and A120F mutant structures of the *ba_3_*-type cytochrome *c* oxidase from *Thermus thermophilus* at 1.8 Å resolution. The enzyme has been crystallized from the lipidic cubic phase, which mimics the biological membrane environment. The structures reveal 20 ordered lipid molecules that occupy binding sites on the protein surface or mediate crystal packing interfaces. The interior of the protein encloses 53 water molecules, including 3 trapped in the designated K-path of proton transfer and 8 in a cluster seen also in A-type enzymes that likely functions in egress of product water and proton translocation. The hydrophobic O_2_-uptake channel, connecting the active site to the lipid bilayer, contains a single water molecule nearest the Cu_B_ atom but otherwise exhibits no residual electron density. The active site contains strong electron density for a pair of bonded atoms bridging the heme Fe_a3_ and Cu_B_ atoms that is best modeled as peroxide. The structure of *ba_3_*-oxidase reveals new information about the positioning of the enzyme within the membrane and the nature of its interactions with lipid molecules. The atomic resolution details provide insight into the mechanisms of electron transfer, oxygen diffusion into the active site, reduction of oxygen to water, and pumping of protons across the membrane. The development of a robust system for production of *ba_3_*-oxidase crystals diffracting to high resolution, together with an established expression system for generating mutants, opens the door for systematic structure-function studies.

## Introduction

Heme/copper oxidases (HCO) represent a superfamily of enzymes found in the aerobic respiratory chain of mitochondria and bacteria that convert oxygen to water and transfer protons across membranes to form an electrochemical gradient. Members of this family include cytochrome *c* and quinol oxidases that have been phylogenetically grouped into three major subfamilies. The A-type is represented by some of the most studied cytochromes *aa_3_* from bovine heart mitochondria [1]–[3] and from two bacteria, *Paracoccus denitrificans* (*Pd*) [4], [5] and *Rhodobacter sphaeroides* (*Rs*) [6], [7]. B-type enzymes, of which the prototypical member is the *ba_3_*-oxidase from *Thermus thermophilus* (*Tt*) [8], [9], are found in both eubacteria and archaea. The C-type oxidases (*cbb_3_*), found in two bacterial groups, are expressed at low oxygen levels, a feature they share with some pathogenic bacteria [10]. While highly divergent in amino acid sequence, HCOs of the different types share common structural elements, suggesting a similar mechanism of action.

Despite decades of investigation and the availability of structures for all HCO types [1]–[10], the details of oxygen reduction and proton transfer are not fully understood. In particular, while computational modeling has recently provided insights into the chemical mechanism of proton pumping in *ba_3_* oxidase, atomic-level, experimental details of the coupling of proton transfer to the chemical steps of O_2_ reduction remain unclear [11]. Similarly, detailed mechanisms of the gating of proton channels to ensure unidirectional proton flow have yet to be elucidated [12]. Finally, questions remain regarding the path for the transfer of O_2_ from the membrane into the heme *a*_3_-Cu_B_ dinuclear center, as well as the nature of the inter-metal O atom(s), observed in the current and previous studies, between Fe_a3_ and Cu_B_ [13], [14].

The *ba_3_* cytochrome *c* oxidase from *T. thermophilus* represents a unique HCO system to study these mechanisms. The non-overlapping optical absorption spectra of the *b*-heme and the *a_3_*-heme centers permit their electronation states to be readily distinguished and quantified [15], [16]. Its low amino acid sequence similarity [17] to the well-studied *aa_3_* oxidases quickly reveals those residues that are evolutionarily conserved and play essential roles in structure and mechanism and those that do not. Additionally, an expression system for *ba_3_* has been developed, allowing for the straightforward generation of mutant enzymes [18], which have already been used to probe details of proton transfer [12], [19] and electron transfer [16] mechanisms.

The crystal structure of *ba_3_*-oxidase solubilized in detergent micelles was previously obtained at 2.4 Å [8] and 2.3 Å [9] resolution; however, while *ba_3_* readily crystallizes in these systems, typically fewer than one crystal out of 30 diffracts better than ∼3 Å, which severely diminishes the possibilities for combined structure-function work at the single crystal level. Toward overcoming this barrier, we applied crystallization in lipidic cubic phase (LCP), also known as *in meso* crystallization [20], [21], and obtained highly reproducible crystals of *ba_3_* oxidase diffracting to 1.7–1.8 Å. Initial success of *in meso* crystallization was related to obtaining high resolution structures of microbial rhodopsins [22]–[27] and recently to revealing several structures of human G protein-coupled receptors [28]–[31]. Apart from paving the way for more reproducible and higher resolution structures, this method also permits lipid content to be manipulated during crystallization trials and specific lipid-protein interactions to be observed [32].

In this work, we expand our understanding of the *ba_3_*-oxidase system along seven lines of enquiry: (1) use of lipidic matrix for HCO crystallization, (2) increasing the diffraction limit from 2.3 Å to 1.8 Å, (3) discovering and mapping evolutionarily conserved lipid binding sites similar to those previously described for A-type enzymes, (4) discovery of a novel lipid binding site extending ∼10 Å out of the plane of the membrane, (5) assignment of a well-resolved Fe_a3_^-^O-O^-^Cu_B_ atomic arrangement indicative of a bound peroxo dianion, (6) new evidence that the large, hydrophobic O_2_-channel is devoid of all but one ordered water, and (7) characterization of a water cluster starting from a single conserved water molecule that bridges the propionate side-chains of heme-*a_3_* and expanding to include 8 water molecules that lie at the interface of subunit I and subunit II.

## Results and Discussion

### Overall protein structure and comparisons with lower resolution *ba_3_* structures

The wild type (WT) protein and A120F mutant of *ba_3_* cytochrome *c* oxidase from *T. thermophilus* were crystallized in lipidic cubic phase and their structures were refined at 1.8 Å (Table 1). The A120F mutant was originally designed to block one of the mid-membrane entrances to the oxygen channel (see ref.13), but it has no effect on activity or the spectral properties of the enzyme; however, it demonstrates a slightly better crystallization behavior. Both structures are almost identical (total RMSD ∼0.29 Å), while the A120F mutant has a better defined electron density with lower B-factors; therefore, the subsequent analysis will be focused on the A120F mutant unless noted otherwise.

**Table 1:** Data Collection and Refinement Statistics.

	A120F	Wild type
PDB code	3S8G	3S8F
Space group	C2	C2
Unit cell dimensions, Å	144.96 98.64 95.06	143.59 97.82 94.95
Unit cell angles, °	90 128.1 90	90 128.3 90
Molecules per asymmetric unit	1	1
Solvent content, %	61.9	61.0
**Data**		
Number of crystals	8	5
Total observations > 0σ_F_	473,696	435,762
Unique reflections > 0σ_F_	95,365	92,466
Resolution, Å	50−1.80 (1.86−1.80)*	50−1.80 (1.86–1.80)
Redundancy	5.0 (3.5)	4.7 (3.4)
Completeness, %	97.8 (92.5)	97.3 (90.2)
<I/σ_I_>	15.1 (2.0)	17.2 (1.7)
Rmerge	0.112 (0.592)	0.097 (0.680)
**Refinement**		
R_work_/R_free_	0.175/0.196	0.187/0.218
Reflections used	90,591	87,794
Test set, 5.0%	4,759	4,656
R.m.s. deviations		
Bond lengths, Å	0.013	0.029
Bond angles, deg.	1.25	2.16
Ramachandran plot		
Favored regions, %	97.9	97.6
Allowed regions, %	2.1	2.1
Disallowed regions, %	0.0	0.3
**Model**	Residues/Avg. B-factors	Residues/Avg. B-factors
Subunit A residues 9–562	554/23.1	554/30.7
Subunit B residues 3–168	166/22.9	166/31.0
Subunit C residues 4–34	31/22.1	31/31.1
Heme *a_3_*	1/15.3	1/22.6
Heme *b*	1/11.8	1/17.0
CuA	1/14.4	1/21.5
CuB	1/15.5	1/23.5
Peroxide	1/15.3	1/20.7
Monoolein	20/62.7	16/64.2
H_2_O molecules	225/31.9	193/35.8

*Values in parentheses are for the highest resolution shell.

The enzyme was crystallized under a number of conditions; however, all crystallization conditions contained a high concentration of PEG400 (40–45%) that swells LCP and converts it into a sponge phase [33], which is consistent with a previous hypothesis that large proteins (>50 kDa) require swelling of LCP in order to have enough room for protein molecules to move and feed into growing crystals [34]. The crystals exhibit type I packing (Figure S1), as in all *in meso* grown crystals to date [35], with one molecule per asymmetric unit in the C2 space group. Each layer in a type I crystal represents a 2D protein crystal with contacts within the layers mediated by protein-lipid or protein-protein interactions and the gaps between protein molecules filled with disordered lipid, forming a lipid bilayer. The protein consists of 3 subunits: a 61.7 kDa core subunit I with 13 transmembrane α-helices (TMHs), containing heme-*b*, heme-*a_3_* and Cu_B_; an 18.5 kDa mostly water soluble subunit II (the Cu_A_ domain) with one TMH, anchoring it to the lipid membrane and to subunit I; and, specific to *ba_3_* oxidase, a 3.8 kDa subunit IIa forming a single TMH, running parallel to the TMH of subunit II.

**Figure 1:**
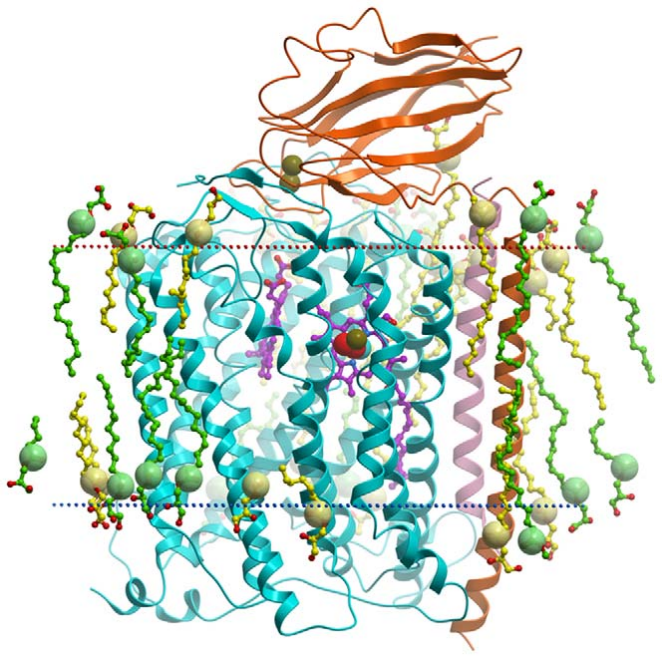
Crystal structure of the *ba_3_* A120F mutant within implicit lipid bilayer. Subunit I is shown in cyan, subunit II is shown in orange, and subunit IIa is shown in pink. Monooleins in the asymmetric unit are shown in yellow and symmetry-related lipids are shown in green, with carboxylic carbons depicted as large spheres. Hemes are shown in magenta. Peroxide, Cu^2+^ and Fe ions are shown by red, brown and cyan spheres, respectively. Red and blue dotted lines indicate periplasmic space and intracellular borders of a hydrophobic slab.

The overall backbone structure of the *ba_3_* oxidase crystallized *in meso* (Figure 1) is nearly identical to that crystallized in detergent micelles [9] (total RMSD between the current structure and PDB ID 1XME [9] is ∼0.38 Å). Differences include the conformation of a weakly ordered loop from Ile512 to Arg518 on the inside rim of subunit I, additional ordered waters detected in the interior of the protein, the absence of the glycerol molecule from an interior cavity as seen in 1XME [9], the presence of two O-atoms, possibly a peroxo dianion, that was previously modeled as a single oxygen atom in 1XME [9], and the presence of ordered lipid molecules on the exterior surface of the enzyme.

The *in meso* structure manifests remarkably low B-factors (Table 1), in contrast to *ba*_3_ oxidase crystallized in detergent micelles where average B-factors are >50 Å [8], [9]. Presumably, this is due to the extensive direct and lipid-mediated crystal contacts involving the transmembrane components, but it also reflects the high degree of order within the protein interior (Figure S2). The new structural elements revealed by the high resolution *in meso* structure include 20 ordered lipid molecules, providing a snapshot of the lipidic environment surrounding *ba_3_* oxidase; 225 ordered water molecules, 53 of which are buried inside the protein, with 29 located in the hydrophilic cavity between subunits I and II where they may be involved in the proton translocation path and the exit route for water molecules from the active site; and a better resolved active site, strongly suggesting placement of a peroxo dianion bound to both the Fe of heme*-a_3_* and to Cu_B_.

### Lipid conformations and lipid-protein interactions

The effects of lipids on membrane protein functions have been extensively reported [36]–[38]. Identification of specific lipid-binding sites on the surface of membrane proteins and their conservation through evolution are concepts just recently beginning to emerge [7], [39]–[42]. Since crystallization of *ba_3_*-oxidase was performed in a lipidic environment, it is expected that the protein molecules would be surrounded by lipids inside the crystal lattice. Examination of electron density revealed a number of strong elongated density tubes aligned around the protein hydrophobic surface, apparently belonging to lipid molecules. Since no specific native lipid head group density was observed [43], all lipids were modeled as monooleins (the LCP host lipid; PDB chemical component code OLC); however, some of them may represent or mimic ordered hydrocarbon chains of tightly bound native lipids, co-purified with the enzyme. Overall, 20 monoolein molecules were modeled in the asymmetric unit. Within hydrophobic layers in the crystal lattice, each protein interacts with three neighbors. After applying symmetry operations, 36 lipid molecules were revealed in the vicinity of each protein, 30 of which are making direct lipid-protein contacts (4 Å cutoff) (Table 2). Ordered lipids cover 3,500 Å^2^ out of the total 10,000 Å^2^ hydrophobic surface of the protein (35%). To date, this structure contains the most complete shell of annular lipids observed for any member of the HCO superfamily. A previous structure of *ba_3_*-oxidase (1EHK [8]) has 3 detergent molecules, while the structures of *aa_3_*-oxidase from *Bos taurus* (*Bt*) (2DYR [3]), *Rs* (2GSM [7]), and *Pd* (3HB3 [5]) contain 13 lipids and 6 detergents (39 fatty acid chains covering 11% of the hydrophobic surface), 6 lipids and 5 detergents (10 fatty acid chains covering 13% of the hydrophobic surface), and 24 detergents (24 fatty acid chains covering 33% of the hydrophobic surface), respectively [3], [5], [7], [8]. For comparison, the most complete lipid shells around membrane proteins were resolved in the structures of bacteriorhodopsin crystallized from lipidic cubic phase (27 lipids per protein trimer covering 79% of the trimer hydrophobic surface, PDB ID 1QHJ [44]), a potassium channel crystallized in a lipid/detergent mixture (64 lipids per protein tetramer, PDB ID 2R9R [45]), and two dimensional crystals of aquaporin AQP0 (36 lipids per protein tetramer, PDB ID 2B6O [46]). The new high-resolution structure of *ba_3_*-oxidase in the lipidic cubic phase offers the opportunity to visualize details of the lipid bilayer and its interaction with this membrane protein.

**Table 2:** Summary of Lipid-Protein interactions.

Lipid	# of resolved hydrocarbons	Z-coord of C1 in bilayer	Lipid surface, Å^2^	Interface with *ba_3_* asym., %	Interface with sym. rel., %	Interface with other lipid, %	Exposed, %	Hydrogen bonds with *ba_3_* and other lipids	B- factor
OLC1	18	14.0	779.4	63	0	41	12		49
OLC2	18	12.4	798.7	31	0	41	36	olc2//o23-olc20//o23	68
OLC3	18	16.2	792.6	23	35	31	24		62
OLC4	16	−10.4	736.1	44	38	49	2	olc4//o25- a/W426/ne1; olc4//o25-a/W341/o; olc4//o23 -a/F213/o	40
OLC5	18	14.2	793.4	31	40	37	14	olc5//o25-b/Y35/oh; olc5//o23 olc8//o25	56
OLC6	18	−19.2	777.5	26	33	28	28	olc6//o23-a/D517/od1;	73
OLC7	18	26.6	788.1	57	0	32	21	olc7//o23-b/E144/o; olc7//o19-b/R141/nh1; olc7//o23-b/R141/nh2	62
OLC8	7	11.5	453.8	43	0	32	40	olc8//o25-olc5//o23	72
OLC9	18	−16.3	801.7	50	5	12	40		59
OLC10	18	19.8	719.5	55	0	22	30		58
OLC11	11	17.0	574.6	37	19	47	19	olc11//o23-olc191//o23	59
OLC12	10	−12.8	551.8	30	20	50	24	olc12//o19-a/W111/ne1; olc12//o19-olc18//o23; olc12//o25-olc18//o25	63
OLC13	1	−13.3	287.2	65	0	16	26	olc13//o25-a/D165/od2; olc13//o19-a/R168/nh1; olc13//o23-a/R168/nh1	54
OLC14	8	−16.7	479.1	55	0	7	41	olc14//o23-a/R168/ne; olc14//o19-a/R168/nh2	55
OLC15	13	17.2	644.4	39	35	49	8	olc15//o19-olc16//o23	58
OLC16	18	15.8	782.6	27	44	47	9	olc16//o23-olc15//o19; olc16//o19-olc3//o23	53
OLC17	14	17.5	667.3	34	36	18	27		60
OLC18	9	−13.1	518.2	45	0	15	46	olc18//o20-a/K19/nz; olc18//o19-a/W111/ne1; olc12//o19-olc18//o23; olc12//o25-olc18//o25	63
OLC19	18	−14.8	778.6	54	0	34	27	olc19//o23-olc111//o23	52
OLC20	5	13.7	406.4	24	0	49	38	olc20//o25-a/W441/ne1; olc20//o23-olc2//o23	68

*Interface with other molecules is calculated as % of the lipid surface masked from solvent by these other molecules. The interfaces with different molecules may partially overlap.

**Figure 2:**
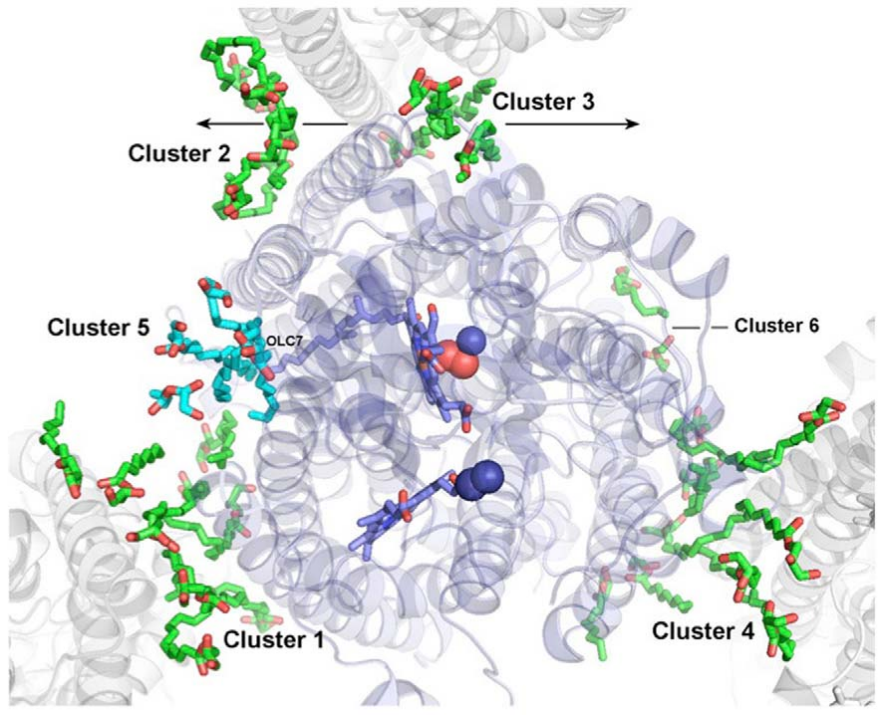
Six clusters of monoolein molecules (green and cyan) surround *ba*_3_ oxidase (ribbon structure) in crystals obtained from the lipidic cubic phase. The copy of *ba*_3_ in the asymmetric unit is light blue while symmetry related molecules are shown in gray. The lipid clusters comprise 34 monooleins, 20 of which are in the asymmetric unit. Clusters 4 and 6 mediate protein-protein packing interactions in the lattice. Clusters 2 and 3 flank an extensive direct protein-protein contact on the crystallographic 2-fold axis (space group C2) that involves the entire length of the N-terminal TM domain of Subunit II (Cu_A_ domain). Clusters 5 and 6 are associated with the protein in the absence of lattice interactions. Cluster 5 overlaps with the region of ordered lipid and detergent binding sites in other cytochrome oxidases, and contains the unique monoolein, OLC7, which extends above the plane of the implicit bilayer (Figure 3A).

All lipid molecules in the current *ba_3_*-oxidase structure can be grouped in 6 clusters (Figure 2, Table S1). Clusters 1 and 4 are symmetry related and mediate protein interactions in the crystal lattice. Clusters 2 and 3 are associated with the direct hydrophobic protein-protein interaction made by subunit II with itself along the two-fold symmetry axis. Clusters 5 and 6 are not involved in any packing interactions, making them perhaps the most physiologically interesting lipids. Most of the lipid chains exist in extended conformations and are aligned perpendicularly to the membrane surface following grooves between α-helices of *ba_3_*. With a few exceptions (e.g. OLC4 and OLC19), the polar head group of the lipid molecules (C24, C22, C21, and C1, with C1 marking the start of the 18-carbon alkyl chain) have the highest B-factor, while the lowest can be found in the middle of the alkyl chain (Figure S3). On average, the lowest B-factor is at C14 of the alkyl chain, about 20% lower than the B-factor of the polar head group. Thus, the properties of lipids composing the annular shell around *ba_3_*-oxidase differ from the properties of lipids in a bulk membrane, in which the probability of forming gauche conformations exhibiting higher B-factors increase steeply with increasing carbon position number in the alkyl chain after the first 6-8 carbons [47].

**Figure 3:**
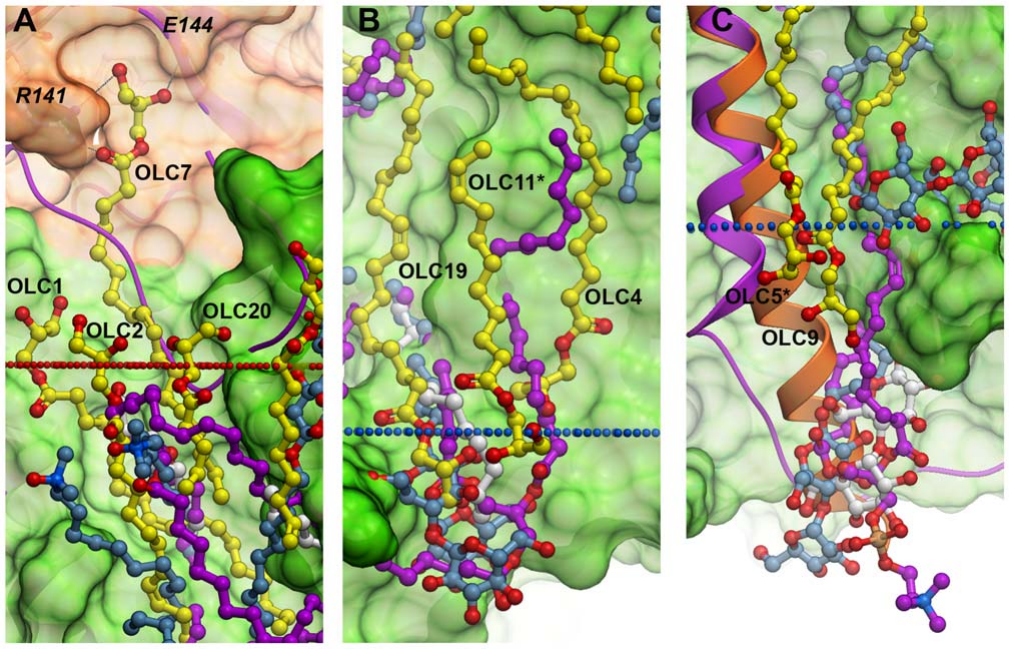
Superposition of lipids associated with the current *ba_3_* structure and lipids associated wtih three highest resolution *aa_3_* oxidase structures. *Ba_3_* structure is shown in green (subunit I and IIa) or orange (subunit II) and *aa_3_* is shown in purple. *Ba_3_* lipids are shown in yellow, lipids of the *Bos taurus* structure (2DYR) in magenta, detergents of the *P. denitrificans* (3HB3) in blue, and detergents of the *R. sphaeroides* (2GSM) structure in white. Labels with an * indicate a symmetry mate. Periplasmic and cytoplasmic membrane boundaries are shown in red and blue dotted lines. (A) Close-up of lipids in cluster 5 occupying a conserved region of lipid binding in cytochrome *c* oxidase and the unique lipid OLC7 that extends out of the membrane, forming specific hydrogen bonds with Arg141 and Glu144. Note that the region occupied by OLC7 in *ba_3_* is filled by the N-terminus of subunit II in *aa_3_* oxidase. (B) Close-up of lipids in cluster 1 occupying a conserved region of lipid binding throughout the cytochrome *c* oxidases. (C) Close-up of a region near cluster 2 that is conserved for lipid binding in *aa_3_-*type oxidases, but not in *ba_3_.* Note that the lipid binding region of the *aa_3_* oxidases is occupied by the end of helix 12 of subunit II of *ba_3_* oxidase (shown in orange), while the equivalent helix in *aa_3_*-type oxidases (shown in purple) does not extend so far.

The hydrophobic thickness of the lipidic membrane surrounding *ba_3_*-oxidase in the crystal structure can be determined from the average positions of the carboxylic groups of the lipid molecules. This thickness was determined to be 30.1 Å, which agrees well with the Orientations of Proteins in Membranes (OPM) predicted value, 31.4±1.3 Å [48]. The standard deviation of lipid positions along the Z-axis from the average membrane plane is 2 Å, which is comparable to the out-of-plane thermal fluctuations of lipids (2.2 Å [49]). The overall tilt of the protein in respect to the membrane normal is only 8±2°, the same as reported by the OPM server [48].

Individual lipid-protein and lipid-lipid interactions for each of the 20 lipid molecules in the asymmetric unit are described in Table 2. All lipids have direct contacts with at least one *ba_3_* molecule, and for seven of the lipids the contact area exceeds 50% of the total molecular surface. Nine lipid molecules are sandwiched between two proteins in the crystal lattice (OLC 3, 4, 5, 6, 11, 12, 17, 18, 19) with a significant interface with both molecules. In addition, most lipids have a substantial interface with other lipid molecules in the crystal lattice, and presumably with unstructured lipid molecules as well.

Fifteen of twenty lipid molecules in the asymmetric unit have at least one hydrogen bond to *ba_3_* or to other lipid head groups in the crystal structure (Table 2). Nine lipid molecules are hydrogen bonded to the protein, while six have polar interactions only with other lipids. Interestingly, some of the strongest protein-lipid hydrogen bonding networks can be observed for those lipid head groups that are significantly shifted out of the membrane plane. For example, OLC4 is pulled ∼5 Å into the bilayer and has strong polar interactions with side chain and main chain atoms of *ba_3_* subunit I, reflected by the low B-factor of the OLC4 head group (Figure S3). Another interesting example is OLC7, for which the head group protrudes ∼10 Å above the extracellular side of the membrane surface and forms strong hydrogen bonds with the side chain of Arg141 (2.8 and 3.2 Å) and the carbonyl of Glu144 (2.8 Å) in a pocket in the soluble Cu_A_ domain (Figure 3A). Eight carbons of the OLC7 hydrocarbon tail are still embedded in the membrane and have tight interactions with other lipid molecules in the structure. The distance between the Cu_2_ atom of Cu_A_ and O23 of OLC7 is ∼20 Å, indicating the absence of a direct functionally important role of this lipid at the Cu_A_ site.

Structural superposition of the *in meso ba_3_*-oxidase structure with known structures of *aa_3_*-oxidases from different species reveals conservation of several lipid binding sites, despite significant differences in protein sequence (Figure S4). A particularly interesting site is centered at cluster 5 (Figure 3A). This region of the protein surface is occupied by the lipid tristearoylglycerol in 2DYR [3], two lauryl dimethylamine-N-oxide detergent molecules in 3HB3 [5], and an ordered tridecane chain in 2GSM [7]. In *ba_3_* oxidase, the lipids in cluster 5 occupy the only concave portion of the protein surface within the membrane and do not participate in crystal packing contacts. This cluster includes two fully ordered molecules, OLC1 and OLC2, as well as the ordered head group of OLC20. OLC1 has distinctly low B-values for its aliphatic tail (Figure S3), and OLCs 1, 2 and 20 surround the translated alkyl chain of OLC7, noted above, which is tethered by strong hydrogen bonding interactions above the plane of the membrane (Figure 3A). Interestingly, while the single TMH of subunit IIa of *ba_3_* terminates near the membrane surface, the spatially equivalent helices in type *aa_3_*-oxidases have protein loops extending from this helix that occupy the same region in space as the lipid chain of OLC7. If the OLC7 site is occupied by a native lipid in *Tt* membranes, where it could interact with Arg141 on subunit II, this positioning of OLC7 could represent an adaptation to the deletion of the homologous protein loop. In particular, a *Tt* lipid in the OLC7 site could help to tether the subunit I and II domains. At the same time, interactions of OLC1 and OLC2 with subunit IIa may stabilize the association of this TMH, which is inverted in orientation relative to the *aa_3_*-oxidases, with subunit I.

Another interesting conserved lipid binding site is in cluster 1 around OLC19, located on the same face of *ba_3_*-oxidase as cluster 5, but directed towards the opposite side of the membrane (Figure 3B). This site is occupied by a well-ordered, dodecyl-β-D-maltoside detergent in 3HB3 and an ordered lipid tail in 2GSM. The head group of OLC19 overlays nicely with the polar portion of the detergent molecule in 3HB3. Several other lipids in *ba_3_* occupy similar sites to lipids or detergents observed in one or more *aa_3_*-structures. OLC16 and OLC3 occupy similar positions to the two alkyl chains of the phospholipid PGV525 in 2DYR. The alkyl chain of OLC3 occupies a very similar position to the alkyl chain of the detergent LMT572 of 3HB3, while a portion of the detergent head group overlaps with the head groups of both OLC3 and OLC16. The conservation of lipid and detergent binding sites in these oxidase structures suggests that lipid binding occurs in response to elements of the protein fold, and not to specific alkyl tail – hydrophobic side chain interactions (Figure S4).

In addition to lipid/detergent binding sites that are conserved between *ba_3_* and *aa_3_*-structures, there are also several sites that are well-conserved among *aa_3_*-structures but are not conserved with *ba_3_*. For example, two adjacent sites bind detergent molecules in 2GSM and 3HB3, while phospholipid tails from PGV occupy very similar sites in 2DYR. One of these two sites has no lipids present in *ba_3_*, potentially because the corresponding TMH α12 (*ba_3_* numbering) with which the lipids interact in *aa_3_*-oxidases is significantly displaced in *ba_3_*_._ The second site contains the head group of a symmetry mate of OLC17, occupying a similar position to the polar moieties on the detergents, but in this case the lipid tail extends in a different direction. Another site that is conserved in all three *aa_3_*-structures contains a detergent molecule in 3HB3 and 2GSM and a phospholipid tail of PSC in 2DYR. Interestingly, this site does not exist in the structure of *ba_3_*: instead, the N-terminus of subunit II extends below the plane of the membrane and occupies the same region in space as the head groups of these lipids (Figure 3C). These examples, and the translation of OLC7 in cluster 5, represent ways in which lipid binding accommodates specific features of membrane protein folds.

### Structured water molecules and their possible roles

**Figure 4:**
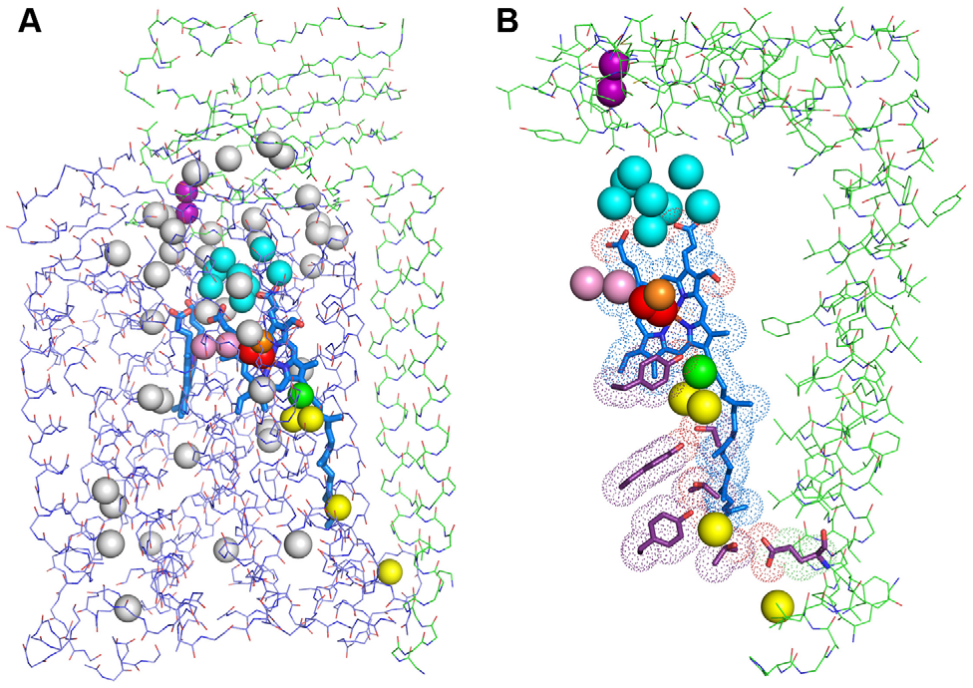
Internal water molecules in the high-resolution structure of *ba_3_* oxidase. (A) Stick representation of main chain atoms of subunits I (blue) and II (green) of *ba_3_*. Water molecules associated with the K-path are shown in yellow; those belonging to the unique cluster (discussed in the text) are shown in cyan; the two water molecules that bridge from Cu_B_ into the Xe1 site in the oxygen channel are shown in light purple; and interior waters not thought to have a functional role are shown in gray. Heme-*a_3_* and heme-*b* are shown in blue sticks, Cu_A_ atoms are shown as dark purple spheres; Cu_B_ is an orange sphere, and the peroxo dianion is shown as red spheres. The secondary OH group of the gernalygeranyl side chain of heme-*a_3_* is shown in green. (B) Close-up view of the internal water cluster (cyan) and K-path. Side-chains of residues of the K-path are shown in purple with a dot surface, and heme-*a_3_* also has a dot surface. The green sphere in the center corresponds to the secondary OH group of the gernalygeranyl side chain of heme-*a_3_,* which also participates in the K-path.

Evidence continues to accrue that water molecules play important roles in cytochrome *c* oxidase function, particularly in the coupling of proton-pumping to the reduction of oxygen at the active site of A-type [50], [51] and B-type [12] enzymes. The asymmetric unit of the current model of *ba_3_*-oxidase contains 225 water molecules, of which 53 are buried inside the protein (Figure 4A). Remarkably, 29 of these interior waters are found within a hydrophilic and highly irregular cavity in the space between the subunits I and II (Figure 4A). Of these 29, 13 are bound to subunit I, and 10 are bound to subunit II. HOH 69 and 93 bridge between subunits I and II, leaving several internal water molecules with no direct linkage to either subunit. In addition, it is likely that there are other water molecules in this region whose positions are not detected under X-ray diffraction, suggesting the presence of a “sea” of waters between the two subunits (see Koepke *et al*. [5] for detailed insight on the possible role of this water cluster in oxidase function).

These 29 water molecules include the semi-conserved cluster above the heme-*a_3_* propionates, a four water cluster (HOHs 26, 64, 103 and 108) at the interface of subunit I and II, and a two water cluster (HOHs 39 and 272) bound exclusively to subunit II. These two small water clusters may serve to pass product water molecules and pumped protons from the internalized water cavity to the external surface of the lipid bilayer, although these may be specific to *ba_3_*. The cluster that lies on top of the heme-*a_3_* propionates contains 8 water molecules (cyan, Figures 4, S5, and S6) anchored by interactions with HOH267, which donates two H-bonds to the propionyl carboxylates of heme-*a_3_* and receives a hydrogen bond from the protonated NE atom of His283, one of the ligands to Cu_B_. HOH267 interacts strongly with HOH265, which in turn interacts weakly with other HOH molecules of the cluster. In this manner, the large cluster extends from the heme-*a_3_*/Cu_B_ binuclear center within subunit I into the water cavity between the subunits, from where, it is reasonable to suggest that it serves to conduct product water molecules and pumped protons out of the protein [5].

Comparison of the new *ba_3_* structure in this region with the two earlier structures of *ba_3_* (1EHK (8) and 1XME (9)) and with that of *Bt* (2ZXW [51]), *Pd* (3HB3 [5]), and *Rs* (2GSM [7]) *aa_3_* enzymes reveal the presence of a similar cluster of 8 to 10 water molecules in which the propionate bridging water is conserved and interacts with one closely situated water that is also conserved (Figure S6). Notably, clusters in *Bt* and *Rs* enzymes include a divalent cation (Mg^2+^ or Mn^2+^), which has, in the case of the *Rs* enzyme, been used to trace the atoms of ^17^O_2_ reduced at the active site [52]. See Figure S6 for stereo representations of each of these structures. Note that glycerol displaces several of the HOH molecules in the 1XME structure, and in the case of *Bt aa_3_*, glycerol is a moderate inhibitor of oxidase activity [53].

The proposed proton-uptake pathway, analogous to the K-path in *aa_3_*-type oxidase structures, contains four water molecules resolved in the new structure (yellow, Figures 4 and S7). The pathway may begin at HOH146, residing at the external end of a small “tube” within the protein that extends ∼9 Å to the conserved Glu15B. The internal portion of this proton pathway corresponds to that identified in lower-resolution structures, with possible proton transfer occurring from the protonated carbonyl group of Glu15B to the deprotonated hydroxyl group of Tyr237A via residues Thr315A, HOH102, Tyr248A, Thr312A, a not-yet-identified water molecule spanning the 4.4 Å from Thr312A to Tyr244A, Ser309A, HOH276, and then to the secondary alcohol of HAS, which interacts with the OH of Tyr237A (Figure S7). Another water molecule, HOH80, is in close proximity to HOH276. The not-yet-observed water molecule was previously postulated [12] as a possible structural element to form an intact K-path capable of Grotthus behavior [54]. The absence of any electron density at this position even at 1.8 Å resolution, however, may suggest a different mechanism of proton transfer between Thr312A and Tyr244A. The extended geranylgeranyl tail of heme a_3_, which runs parallel to the K-path, may serve to stabilize burial of nearby polar side chains and water molecules.

Several of the remaining interior water molecules are involved in hydration of the hemes. HOH71 interacts with the heme-*b* (HEM) propionate atoms O2A and O2D and with the NE atom of Arg449A. HOH85 also interacts with O2A of heme-*b*, and HOH82 interacts with O1D of heme-*b*. Additional solvation of the heme-*b* propionates involves Arg450. HOH267 interacts with heme-*a_3_* (HAS) propionate atoms O2A and O2D and additionally with the ND atom of the Cu_B_ ligand, His283A (Figure S5). Solvation of the D-ring propionate of heme-*a_3_* is completed by interaction of its O2D atom with HOH87 (Figures 4B and S5). There are no other water molecules in the vicinity of heme-*a_3_*. The remaining interior waters exist as isolated single or interacting pairs of water molecules that give no indication of involvement in oxidase function, although some of these HOH may be remnants of historical evolution during which the D-channel was lost [12] or possibly pioneer sites of an evolving D-channel.

### The oxygen uptake channel

The proposed oxygen channel of *ba_3_* is a continuous, 18–20 Å long, Y-shaped channel lined by hydrophobic residues leading from the membrane-facing surface of the protein into the heme-*a_3_*-Cu_B_ center (previously described in detail [13]). The new structures confirm earlier data, which suggested that there are no structural waters in the channel [13]. With the exception of one water molecule, HOH165, found at the previously-characterized Xe1 binding site [13] (Figure S8), the 1.8 Å resolution maps show no residual electron density in the Y-shaped channel. The A120F mutation, which was designed with the hope of blocking one of the two entrances to the oxygen channel, is isomorphous with wild type with the Phe120 side chain occluding the entrance to the channel (Figure S8). By itself, this mutation has no effect on the oxidase activity of the mutant form (data not shown), suggesting that the two entrances are redundant and the enzyme can fully function with one of them blocked.

A chain of four oxygen atoms at the active-center end of the channel is comprised of the peroxo dianion (red; PRX O2, see below) bound to Cu_B_ and two water molecules (purple) (Figure S8). The chain includes HOH65, which lies 3.0 Å from the Cu_B_-bound peroxo oxygen atom and makes weak contact with the plane of the porphyrin at HAS C3A. In turn, HOH65 hydrogen bonds with HOH165. Both HOH65 and HOH165 have potential hydrogen bonds with the carbonyl of Gly232A (see Figures S8 and S9), which was earlier shown to be part of the O_2_-channel in the *Tt* enzyme (see supplementary material of Ref. [13]). HOH65 resides between the innermost Xe site (Xe1) and the O-atom of the peroxo dianion coordinated to Cu_B_. The *Tt* HOH65 water binding site is in the same position as the HOH6601 water binding site associated with Gly283 in the *Rs* enzyme [7], and this region of the active site superposes very well in the two proteins. In the *Tt* structure, HOH65 is above the heme and is flanked by the carbonyl of Gly232, Trp229, and by His283, while in the *Rs* structure HOH6601 is flanked by the carbonyl of Gly283, Trp280, and His334. Water at position 165 is unique to *Tt* because it occupies the Xe1 site where it forms a H-bond to the carbonyl of Gly232 (3.4 Å) and to HOH65 (2.64 Å). There is no corresponding HOH in the *Rs* structure, possibly because the phenol OH of Tyr133 in the *Tt* structure is replaced with Trp172 in the *Rs* structure, the side chain of which is flipped up into the O_2_ channel where it appears to occlude a potential water binding site. It is of considerable interest that the Gly283Val mutation in *Rs* strongly inhibits access of O_2_ into the active site [55], although a structure of this mutation has not been reported. Future analyses of conserved water positions throughout the known structures are likely to shed further light on water function in the enzyme.

The previously characterized Xe1 binding site comprises numerous hydrophobic atoms that can contact a xenon atom or a water molecule if present (see supplementary material of Ref. [13]). Alignment of the Xe bearing structure, PDB code 3BVD [13], with our final structural model places HOH165 within the Xe1 site. With respect to the 17 atoms that might interact with Xe1, (see supplementary material of Ref.[13]) the average displacement between Xe1 in 3BVD and HOH165 is only 0.35 Å. It is reasonable to conclude that HOH165 has entered the Xe1 site, most likely from the opposite direction of Xe or O_2_ molecules. The |*F_o_*| *-* |*F_c_*| electron density maps provide the first experimental evidence that one of the Xe binding sites can bind water, but there is no evidence, even for partial occupation, of the remaining Xe-binding sites. Hence, the remainder of this large hydrophobic cavity may be empty (see Ref. [56]).

### The redox active sites

The *ba_3_*-oxidase, like all cytochrome *c* oxidases, contains four metal-based, redox-active sites. In the new structure, the metrics of the two-copper containing Cu_A_ center, situated in subunit II (see Figure 1) and the heme-*b* are highly similar to those of previous reports (PDB codes 2CUA, 1EHK, and 1XME). The Cu_A_ receives electrons from cytochrome *c_552_* while heme-*b* receives electrons from Cu_A_ and donates them to the dioxygen reduction site composed of Cu_B_ and the high-spin heme *a_3_* [16]. While the overall structure from the new crystals is not substantially different than the previously-determined structures at 2.3 Å (PDB code 1XME [9]) and at 2.4 Å (PDB code 1EHK [8]), those studies showed that the structure of the heme-*a_3_*/Cu_B_ site is affected by temperature and time of exposure to the X-ray beams that typify synchrotron sources (see [14], references therein and unpublished work).

**Figure 5:**
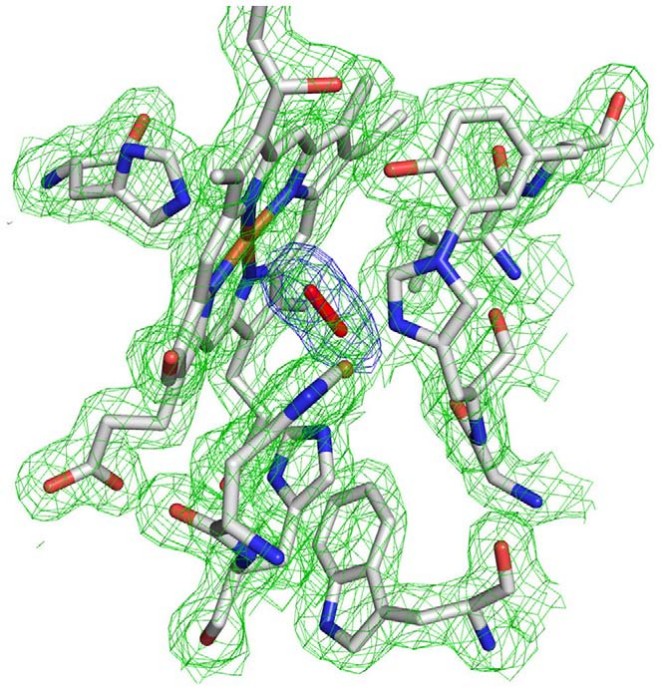
Electron density around active site. 2F_o_-F_c_ electron density is shown in green mesh at 1.5σ. The unbiased F_o_-F_c_ difference density is shown in blue at 3.5σ.

The distance between the iron of heme-*a_3_* and the NE-atom of the proximal His384A is 2.2 Å, compared to 3.3 Å in 1EHK and 2.5 Å in 1XME. These are trending towards shorter Fe-N bonding with higher resolution, as seen with the reported distances in *Bt aa_3_* (1.9 Å, PDB code 2DYR [3]), *Pd aa_3_* (2.1 Å, PDB code 3HB3 [5]), and hemoglobin (1.98 Å) [57]. Another difference is the proximity of the Cu_B_ to the iron of heme-*a_3_*, with a distance of 4.9 Å in the current structure compared to 4.4 Å reported at lower resolution (1XME). This rather large difference is consistent with the idea that the space between Fe_a3_ and Cu_B_ is able to fluctuate in response to ongoing chemistry [14]. Other inter-atom distances between the redox centers are comparable if not identical in the three models (Table S2). However, a major difference at the active site of the new structure of *ba_3_*, compared to previous structures, is the modeling of a peroxide ion bridging the iron of heme-*a_3_* and Cu_B_. In previous studies (1EHK and 1XME) the electron density in this region was spherical and best, but not well, fit with a single oxygen atom ∼2.3 Å from both iron and Cu_B_. In the current omit map of this region the density is elongated (Figure 5). Refinement was performed in several ways, modeling: (i) a single water, (ii) two waters with partial occupancy, (iii) two O-atoms separated by a fixed distance of 1.47 Å, and (iv) two unrestrained O-atoms. The latter model best fits the density, refining to an O-O distance of 1.52 Å (within error of an expected 1.47 Å distance between the two oxygen atoms in a metal bound, peroxo dianion [58]). The O-metal distances of 2.39 Å to heme *a_3_*-Fe and 2.25 Å to Cu_B_ are consistent with other structures. In the *aa_3_*-type oxidases, a similar metal-bridging ligand has been variously modeled as a single oxygen atom or a peroxide in *Rs aa_3_*, 2GSM [7] and in *Pd aa_3_,* 1AR1 [59], as an O-O model with distance of 1.7 Å in *B t aa_3_,* 2ZXW [51], and as a two oxygen atom arrangement which refined to a distance of 1.64 Å in *Pd aa_3_* [5]. In this recent structure of *Pd aa_3_* (3HB3 [5]), careful refinement with several alternative possibilities led the authors to conclude that the electron density in this region probably represents a peroxo dianion (O-O distance 1.49 Å), and the current structure would support that idea.

While care should be taken not to impart mechanistic meaning to these observations, there can be little doubt that two O-atoms 1.5 Å apart are in a bonding situation, and the most likely electronic state is that of peroxide, although the protonation states of the O-atoms can only be guessed (see, however, Ref. [60]). Spectroscopic support for a bridging peroxide in the oxidized state of the bovine heart enzyme comes from the work of Sakaguchi *et al*. [61] who demonstrated the presence of a resonance Raman band at ∼755 cm^-1^ with 647.1 nm excitation into the 650 nm band of the oxidized protein. Such a frequency is compatible with a peroxide bond length of 1.49 Å; the reported O-O distance of 1.7 Å [51] should thus be reconsidered (see Ref. [60]).

Time-resolved, resonance Raman studies of oxygen reduction have failed to identify a peroxide intermediate (see [11] for references) in the reaction pathway of *ba_3_*. Indeed, such an intermediate almost certainly exists, but it must also have an extremely short lifetime, and would not be expected to be trapped, even at quite low temperatures [62]. Most likely, the putative peroxide is formed as a result of X-ray radiation to which the crystals are exposed during data collection. The crystals are formed from the as-isolated enzyme at room temperature, in which spectral properties indicate all the redox cofactors are oxidized. Under this condition no electrons are available to reduce O_2_, present at ∼1 mM concentrations, to H_2_O_2_. The crystals are exposed to X-rays only after freezing at ∼100 K. Therefore, if peroxide is bound in the active site, as the observations suggest, it is most likely formed from atoms present in or very close to the oxidized Fe_a3_/Cu_B_ site after being exposed to X-ray radiation (see, however, Footnote 13 of Ref. [2], and Ref. [51] for contrary opinions).

In addition to high-energy X-ray radiation (12 keV) the crystals are also exposed to a flux of H• and OH•, the primary products of water radiolysis. H• transfers an electron to the medium, forming a proton and the hydrated electron, the latter of which has a lifetime in the microsecond range and is highly mobile, even in frozen water. Most likely, it is responsible for reduction of the redox sites in *ba_3_* during collection of X-ray diffraction data [14]. Although we have not recorded optical absorption spectra of our crystals after irradiation, the Cu_A_, heme-*b*, and heme-*a_3_* sites are undoubtedly reduced. We speculate that the bridging peroxide observed in our data might arise from the recombination of two radiation produced OH• radicals formed either very near to or even in the space between the two metals of the active site.

The orientation of the peroxide between the Fe- and Cu-atoms is different in *Tt ba_3_* than in *Pd aa_3_* (3HB3 [5]), but it is similar to that observed for the putative peroxide in *Bt aa_3_* (3ABL and 2ZXW [51]) (Table 3). In the structure of *Pd aa_3_* (3HB3), the peroxide ion is located just 1.9 Å from the Fe and Cu_B_, and the dihedral angle of heme-*a_3_*-O-O-Cu_B_ is +175°. The equivalent angle in the current structure of *ba_3_* is −147° (−146.9° in *Bt* 3ABL), providing a significantly different orientation of the peroxide relative to the plane of heme-*a_3_*_._ A different product from this type of chemistry might be expected if the two putative water molecules were to reside at different positions within the active sites of the different enzymes prior to exposure to X-ray radiation. Whatever the cause, the physiological relevance of this peroxide remains unclear.

**Table 3:** Summary of observed peroxide geometry.

Species	O−O	Fe−O	O−Cu	(H−O−O) Fe−O−O	(H−O−O−H) Fe−O−O−X
H_2_O_2_	1.47 Å	NA	NA	95°	+120°
Mb-OOH, 2Z6S [68]	1.33 Å	1.85 Å	NA	120°	NA
CPO-OOH, 2J5M [69]	1.50 Å	1.80 Å	NA	131°	NA
Fe−O−O−Cu_B_, *Tt ba_3_, current data*	1.52 Å	2.39 Å	2.25 Å	140°	−147°
Fe−O−O−Cu_B_, *Bt aa_3_,* 2ZXW [51]	1.70 Å	2.23 Å	2.08 Å	154°	−141°
Fe−O−O−Cu_B_, *Bt aa_3_,* 3ABL [51]	1.70 Å	2.23 Å	2.17 Å	144°	−147°
Fe−O−O−Cu_B_, *Pd aa_3_,* 3HB3 [5]	1.49 Å	1.93 Å	1.92 Å	108°	+175°

### Conclusions

We have described a highly-refined, high-resolution structure of cytochrome *ba_3_* oxidase from *T. thermophilus* from which the following conclusions could be drawn. When crystallized in the lipidic cubic phase, 20 lipids are found surrounding the protein in the asymmetric unit and their interactions with the protein are elucidated. Among these are lipid-protein interactions found in previous structures of *aa_3_-*type oxidases that appear to be conserved. One lipid, OLC7, has a previously-unobserved interaction with the water soluble portion of subunit II, lifting the lipid partially out of the lipid bilayer and suggesting that the surface of the lipid bilayer, in close proximity to the protein surface, may be highly irregular. The observed water molecules, most of which are associated with subunit II and the hydrophilic interface between subunits I and II, include a cluster of 8 water molecules. Among this cluster, a previously recognized water molecule bridges the two propionates of heme-*a_3_* and interacts strongly with a second conserved water that traverses the subunit I/subunit II surfaces. In turn, the latter HOH has access to the remaining waters of the cluster that lie outside subunit I. Such a cluster appears to be a conserved feature of A- and B-type cytochromes *c* oxidases and is likely to be important in both water egress and proton pumping. Finally, the observed active-site density is best interpreted in terms of two O-atoms separated by 1.5 Å, likely a peroxo dianion, that bridges from Cu_B_ to Fe_a3_. This feature has been observed in other oxidase structures with varying degrees of clarity. A mechanism for its formation under X-ray radiation is suggested in which two HO• recombine (2 HO• → H_2_O_2_) to form peroxide within the active site. This entity is not likely to be of physiological importance. The development of a crystallization system for *ba_3_* oxidase capable of reliable production of crystals diffracting to 1.8 Å or better, in combination with the previously reported expression system for the straightforward generation of *ba_3_* mutants [18], opens the door for future structure-function studies at the single-crystal level.

## Materials and Methods

### Expression and purification

Recombinant *ba_3_* cytochrome c oxidase was expressed in *Thermus thermophilus* cells and purified as previously described [18]. The protein was concentrated to 10–15 mg/mL in 1 mM dodecyl-β-D-maltoside detergent solution, and stored at 4°C until used.

### Crystallization

Before starting crystallization trials protein solution was spun at 15,000 g for 10 min at 4°C. After spinning, protein was reconstituted in a lipidic cubic phase (LCP) by combining monoolein (Sigma) and protein solution at 3/2 v/v ratio and homogenizing them with a syringe mixer [21], [63]. Crystallization trials were performed in 96-well glass sandwich plates by an *in meso* crystallization robot [64] using 50 nL protein-laden LCP (lipidic cubic phase) overlaid with 0.8 µL precipitant solution in each well, and sealed with a glass coverslip. Protein reconstitution in LCP and crystallization trials were carried out at room temperature (∼21–23°C). The crystallization plates were stored and imaged in an incubator/imager (RockImager 1000, Formulatrix) at 20°C. Diffraction quality crystals of an average size of 60×50×25 µm were obtained within 14 days in 40–45% (v/v) PEG 400, 1.0 to 1.6 M sodium chloride, 100 mM sodium cacodylate trihydrate pH 5.5–6.5. Crystals were harvested using 50–100 µm nylon loops and immediately flash frozen in liquid nitrogen without adding an extra cryoprotectant.

### X-ray data collection and processing

Crystallographic data were collected on the 23ID-B beamline (GM/CA CAT) at the Argonne National Laboratory using a 20 µm collimated minibeam at a wavelength of 1.0330 Å and a MarMosaic 300 detector. To reduce radiation damage crystals were translated to a fresh position, if possible, or replaced after collecting 20 frames at 1 s exposure and 1° oscillation with an unattenuated beam. Datasets were integrated, scaled and merged together using HKL2000 [65]. The wild-type recombinant structure (PDB code 1XME) was used for molecular-replacement calculations with *Phaser* [66]. The resulting model was refined using Refmac5 and repeated rounds of model adjustment using the ΣA-weighted 2|F_o_|-|F_c_| and |F_o_|-|F_c_| electron density maps visualized using MiFit [67]. The coordinates and structure factors of the WT enzyme and of the A120F mutant have been deposited to the Protein Data Bank with accession numbers 3S8F and 3S8G, respectively. Figures were created using *PyMOL* (http://www.pymol.org) and *ICM* (Molsoft).

## Supporting Information

Figure S1 **Type I crystal packing as observed with** ***in meso*** **grown crystals of** ***ba_3_*** **.** *Ba_3_* chains are shown in gray cartoon, the active site hemes are shown in blue sticks, and lipid molecules are shown as green sticks (O atoms in red). Note the alternating orientation of *ba_3_* molecules in the crystal and the obvious layers.
(TIF)

Figure S2 **Distribution of thermal displacement B-factors in the** ***ba_3_*** **structure.** Structure is colored according to B-values (blue: low to red: high). Notice how the interior of the structure is highly ordered and the highest B-values are observed for the exterior lipid molecules.
(TIF)

Figure S3 **Distribution of B-factor values along hydrocarbon chain of lipid molecules.** Head group (glycerol) carbons are numbered C21, C22, C24. The mean value for each carbon position across all 20 lipid molecules is shown by black squares.
(TIF)

Figure S4 **Structure-based sequence alignment of conserved lipid binding sites in** ***ba_3_*** **.** Alignment is performed between the structures of *Tt ba_3_* (this work), *Bt aa_3_* (2DYR), *Rs aa_3_* (2GSM) and *Pd aa_3_* (3HB3). Although several residues (W426, F429 and H432) are conserved in some of the sites, they form non-specific, non-polar contacts with lipid chains
(TIF)

Figure S5 **A cluster of eight internal water molecules (cyan) that interact with the heme-** ***a_3_*** **propionates and residues of subunits I (blue) and II (green).** HOH267 and HOH265 are conserved in other types cytochrome *c* oxidases and are likely to participate in the transport of product water molecules and pumped protons away from the catalytic center. The hydrophilic cavity is surrounded by polar side chains or main chain atoms of 13 residues, and each of the waters has at least one hydrogen bond with the protein. There is no remaining volume inside the cavity to accommodate additional water molecules. The peroxo dianion is shown in red.
(TIF)

Figure S6 **Stereo visual structural comparison of the inter-subunit water cluster in four different enzymes.** The new *ba_3_* structure (upper left); 1XME, *ba_3_* with glycerol; 1EHK, *ba_3_* original; 2ZXW, Bovine *aa_3_*; 2GSM, *Rhodobacter sphaeroides aa_3_*; and 3HB3, *Paracoccus denitrificans aa_3_*.
(TIF)

Figure S7 **The proposed proton-uptake pathway (K path) in** ***ba_3_*** **oxidase linking the cytosolic surface of the protein to the active center.** Side chains of participating residues in subunits I and II (chains A, B) are purple, linking water molecules are yellow, and the secondary alcohol of the heme-*a_3_* side chain is green. Oxygen atoms in the pathway are within hydrogen bonding distance of their nearest neighbors; the gap between Thr312A and Tyr244A (4.4 Å) is expected to be occupied by a not yet resolved water molecule. HOH80 is also within a hydrogen bonding distance to HOH276, Ser309A, and the secondary alcohol. HOH146 at the cytosolic surface of the protein can communicate with Glu15B, a strictly conserved residue, via a ∼9 Å long, loosely packed tube. Water molecules may also access Glu15B via Lys16B at the surface of the subunit II N-terminal transmembrane helix.
(TIF)

Figure S8 **Comparison of the Y-shaped oxygen channel in the wild type** ***ba_3_*** **and in the A120F mutant structures.** The channel (green transparent surface) connects the active site of the enzyme with the protein- lipid interface. While in the WT structure both branches of the Y-shaped channel are opened to the surface of the protein (A120 shown in cyan sticks), the F120 side chain in the mutant protein (black sticks) completely blocks one of the openings.
(TIF)

Figure S9 **A close up of the active center.** A chain of four oxygen atoms in the active center includes the peroxo dianion (red), coordinated to the Fe atom of heme-*a_3_* (HAS) and the Cu_B_ atom (orange), and two water molecules, HOH65 and HOH165 (purple). HOH165 resides in the Xe1 site, i.e. an expected O_2_ binding site in the oxygen diffusion channel, nearest to the active center. HOH65 is hydrogen bonded to both the peroxo dianion and HOH165, and both water molecules can form hydrogen bonds with Gly232A in the oxygen diffusion channel. The hydrogen bond between the secondary alcohol of the heme-*a_3_* side chain (green) and Tyr237A represents the terminus of the proton-uptake pathway (Figure S7).
(TIF)

Table S1 **Assignment of lipids to clusters.**
(PDF)

Table S2 **Comparison of ligand geometry to previous** ***ba_3_*** **structures.**
(PDF)
