# Supplementary figures and images for: High Resolution Structure of the ba3 Cytochrome c Oxidase from Thermus thermophilus in a Lipidic Environment
Source: PLoS One. 2011 Jul 21;6(7):e22348. doi: 10.1371/journal.pone.0022348 (PMC3141039; doi:10.1371/journal.pone.0022348)

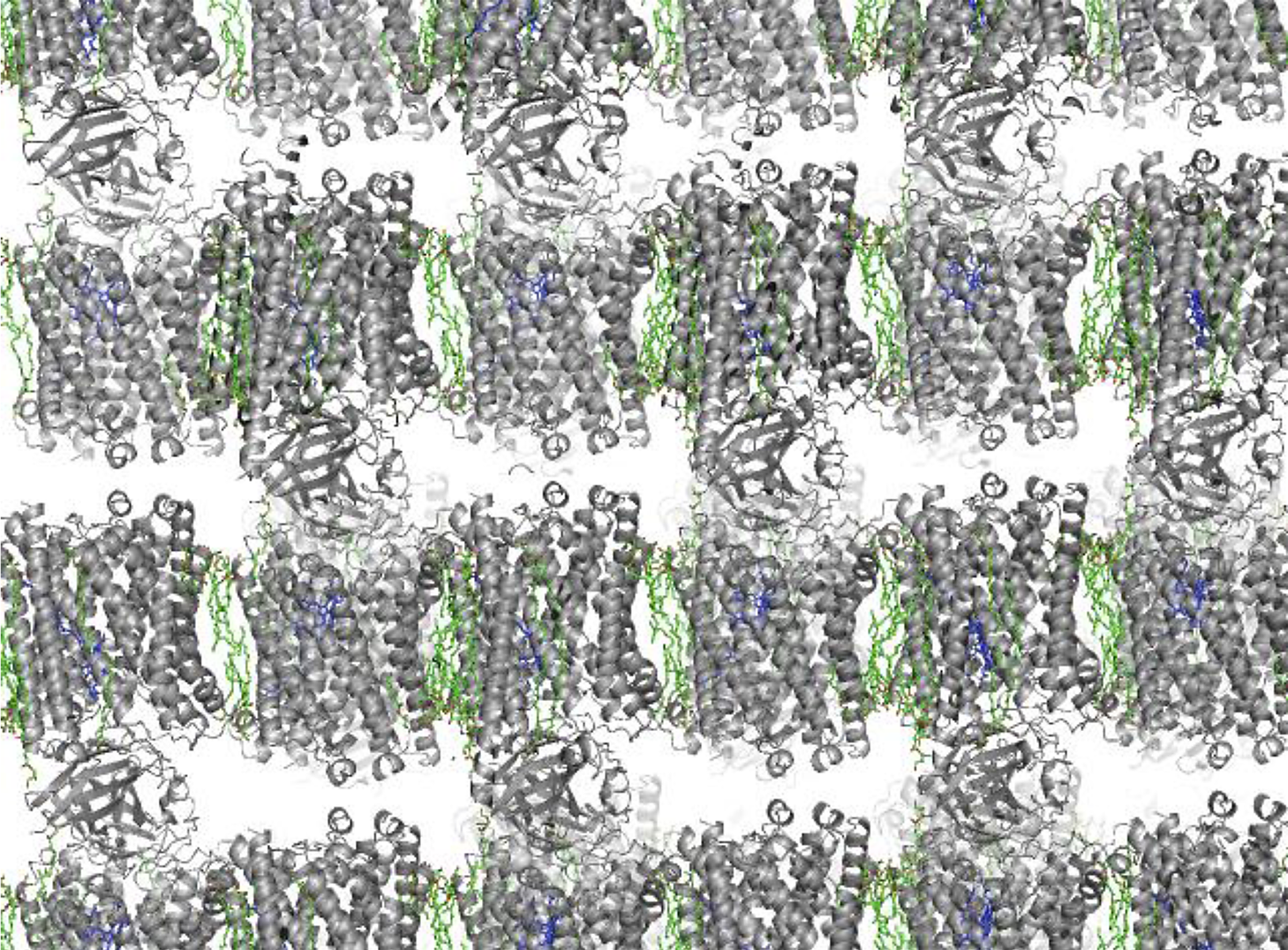

Supplement: Figure S1 — Type I crystal packing as observed with in meso grown crystals of ba3 . Ba3 chains are shown in gray cartoon, the active site hemes are shown in blue sticks, and lipid molecules are shown as green sticks (O atoms in red). Note the alternating orientation of ba3 molecules in the crystal and the obvious layers. (TIF) [file pone.0022348.s001.tif]

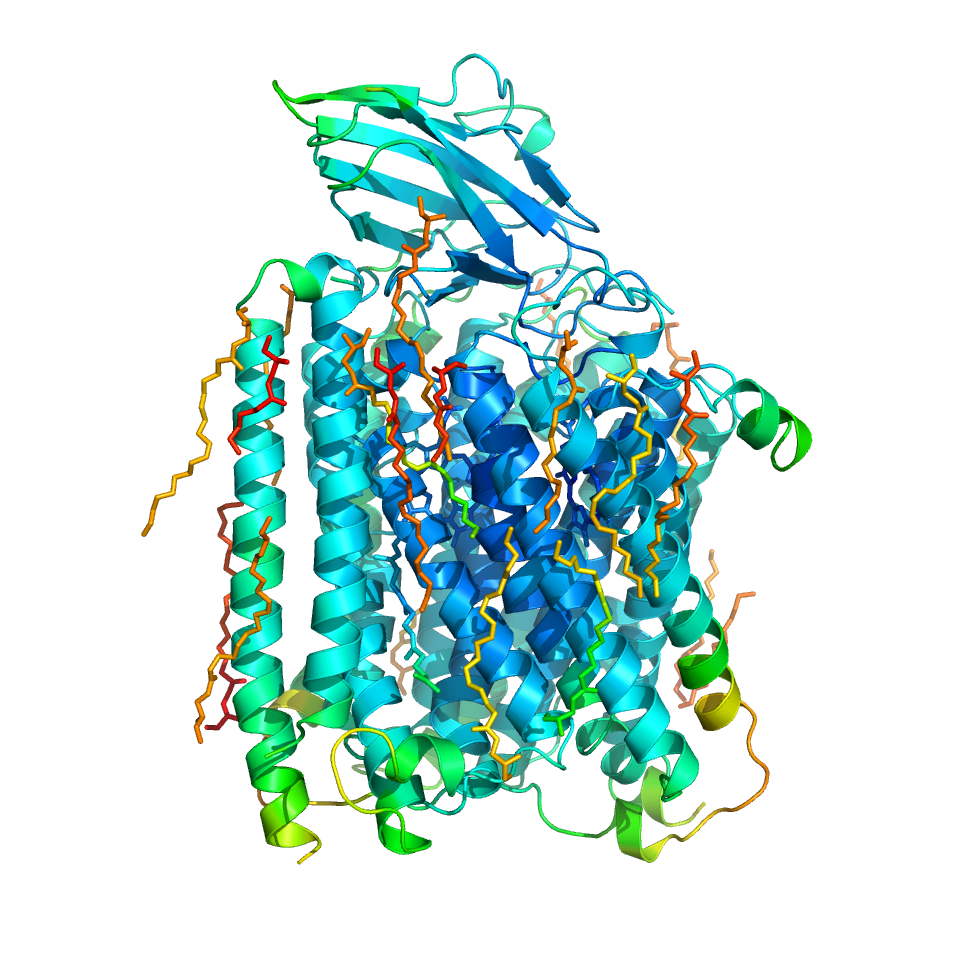

Supplement: Figure S2 — Distribution of thermal displacement B-factors in the ba3 structure. Structure is colored according to B-values (blue: low to red: high). Notice how the interior of the structure is highly ordered and the highest B-values are observed for the exterior lipid molecules. (TIF) [file pone.0022348.s002.tif]

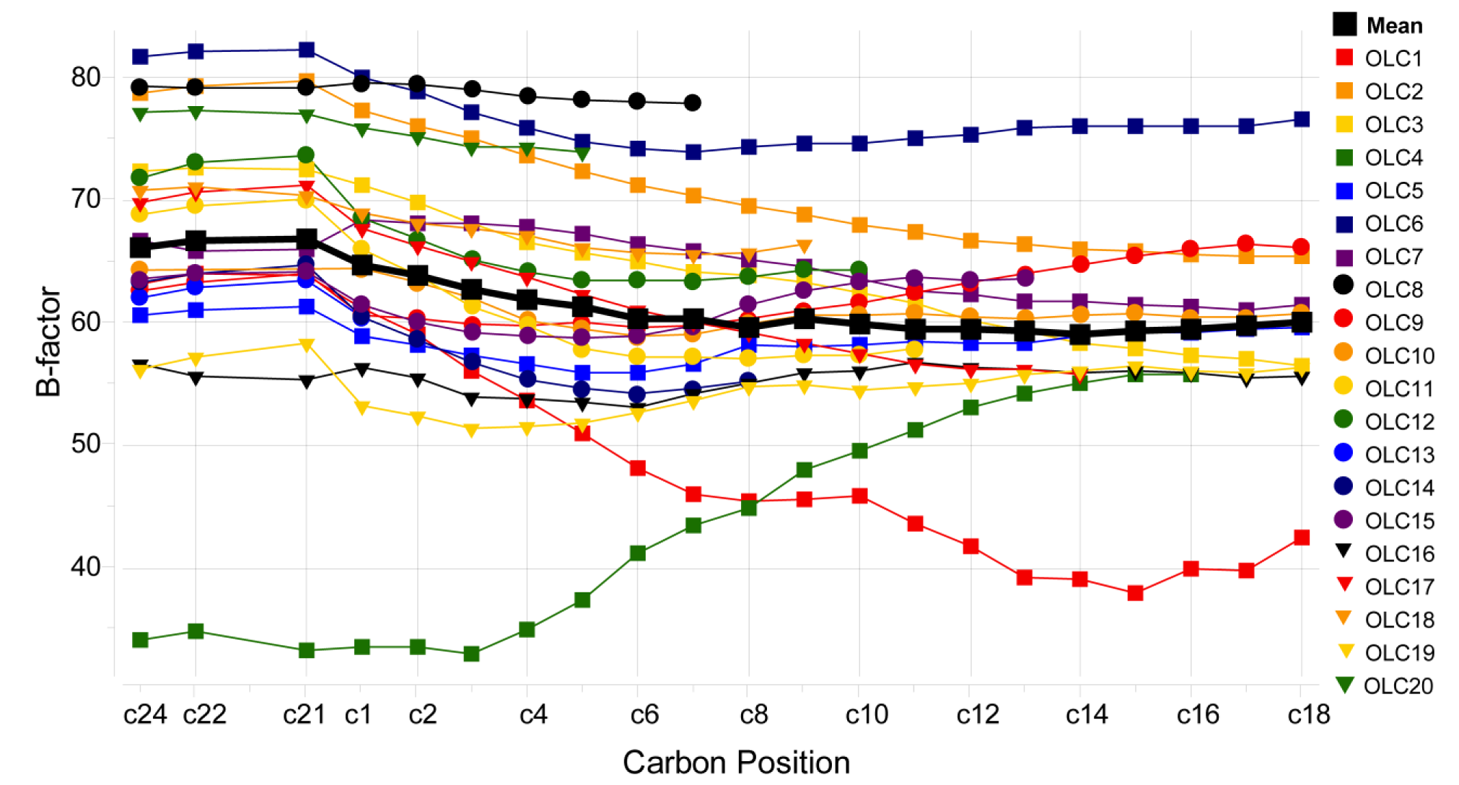

Supplement: Figure S3 — Distribution of B-factor values along hydrocarbon chain of lipid molecules. Head group (glycerol) carbons are numbered C21, C22, C24. The mean value for each carbon position across all 20 lipid molecules is shown by black squares. (TIF) [file pone.0022348.s003.tif]

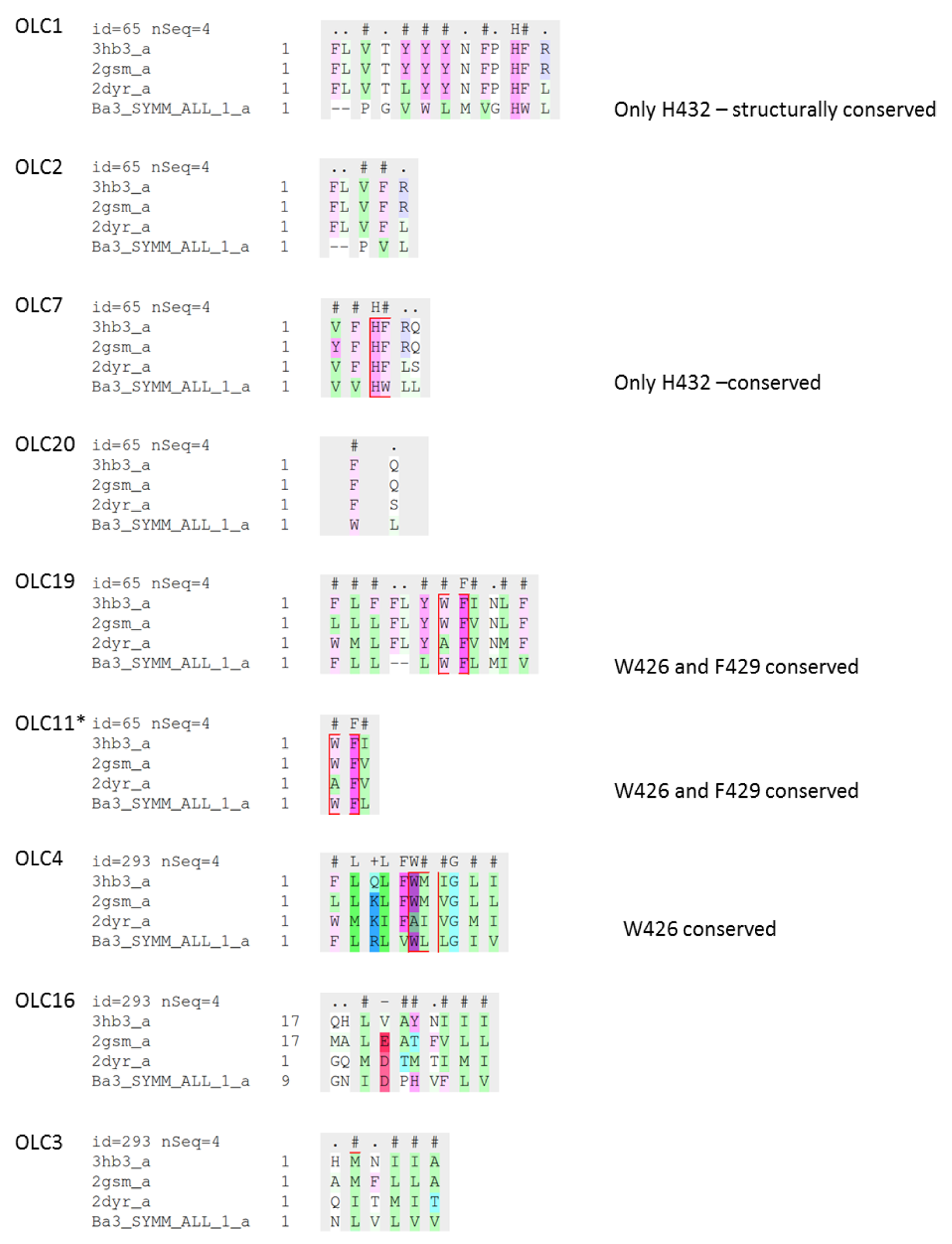

Supplement: Figure S4 — Structure-based sequence alignment of conserved lipid binding sites in ba3 . Alignment is performed between the structures of Tt ba3 (this work), Bt aa3 (2DYR), Rs aa3 (2GSM) and Pd aa3 (3HB3). Although several residues (W426, F429 and H432) are conserved in some of the sites, they form non-specific, non-polar contacts with lipid chains (TIF) [file pone.0022348.s004.tif]

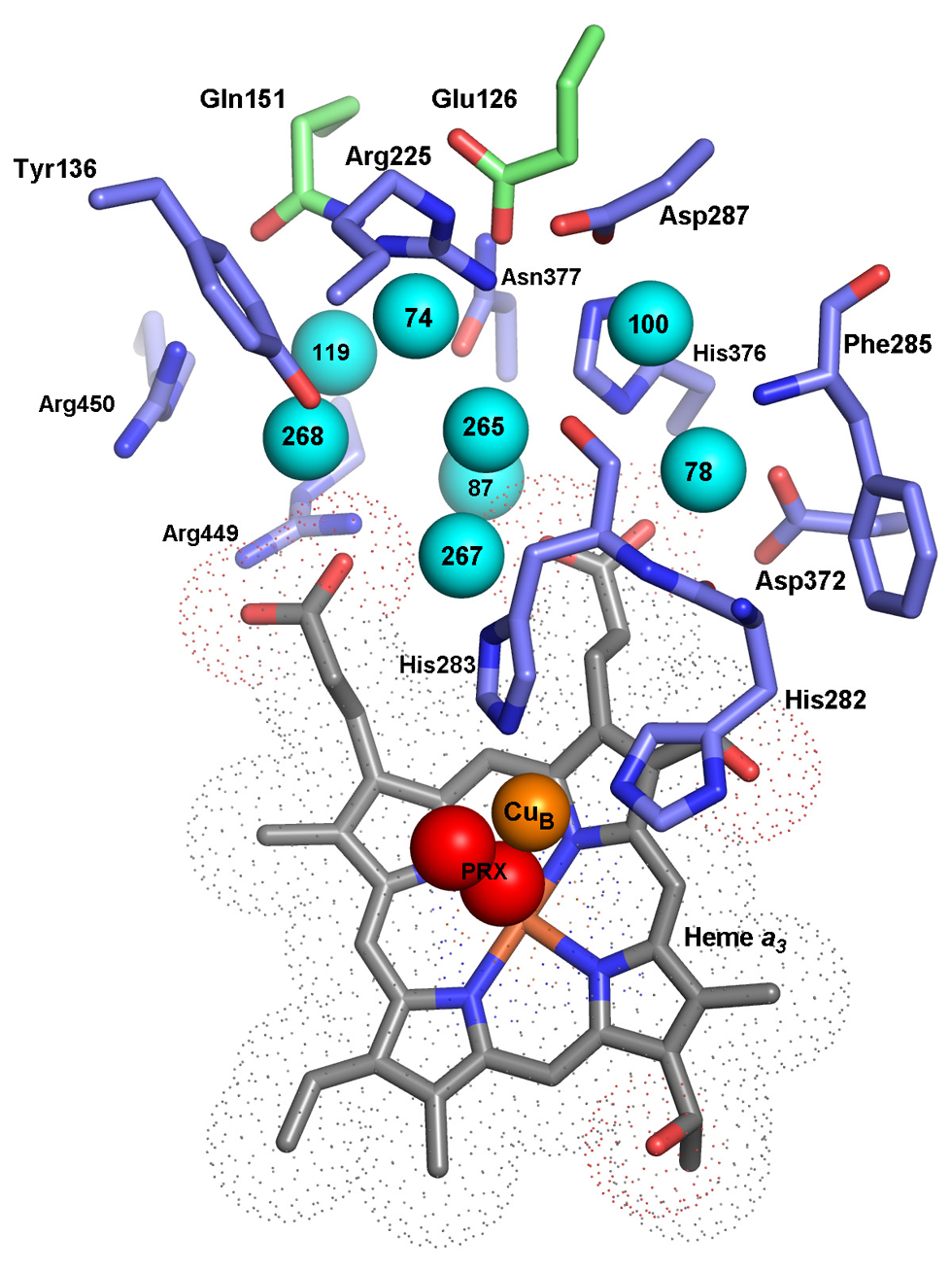

Supplement: Figure S5 — A cluster of eight internal water molecules (cyan) that interact with the heme- a3 propionates and residues of subunits I (blue) and II (green). HOH267 and HOH265 are conserved in other types cytochrome c oxidases and are likely to participate in the transport of product water molecules and pumped protons away from the catalytic center. The hydrophilic cavity is surrounded by polar side chains or main chain atoms of 13 residues, and each of the waters has at least one hydrogen bond with the protein. There is no remaining volume inside the cavity to accommodate additional water molecules. The peroxo dianion is shown in red. (TIF) [file pone.0022348.s005.tif]

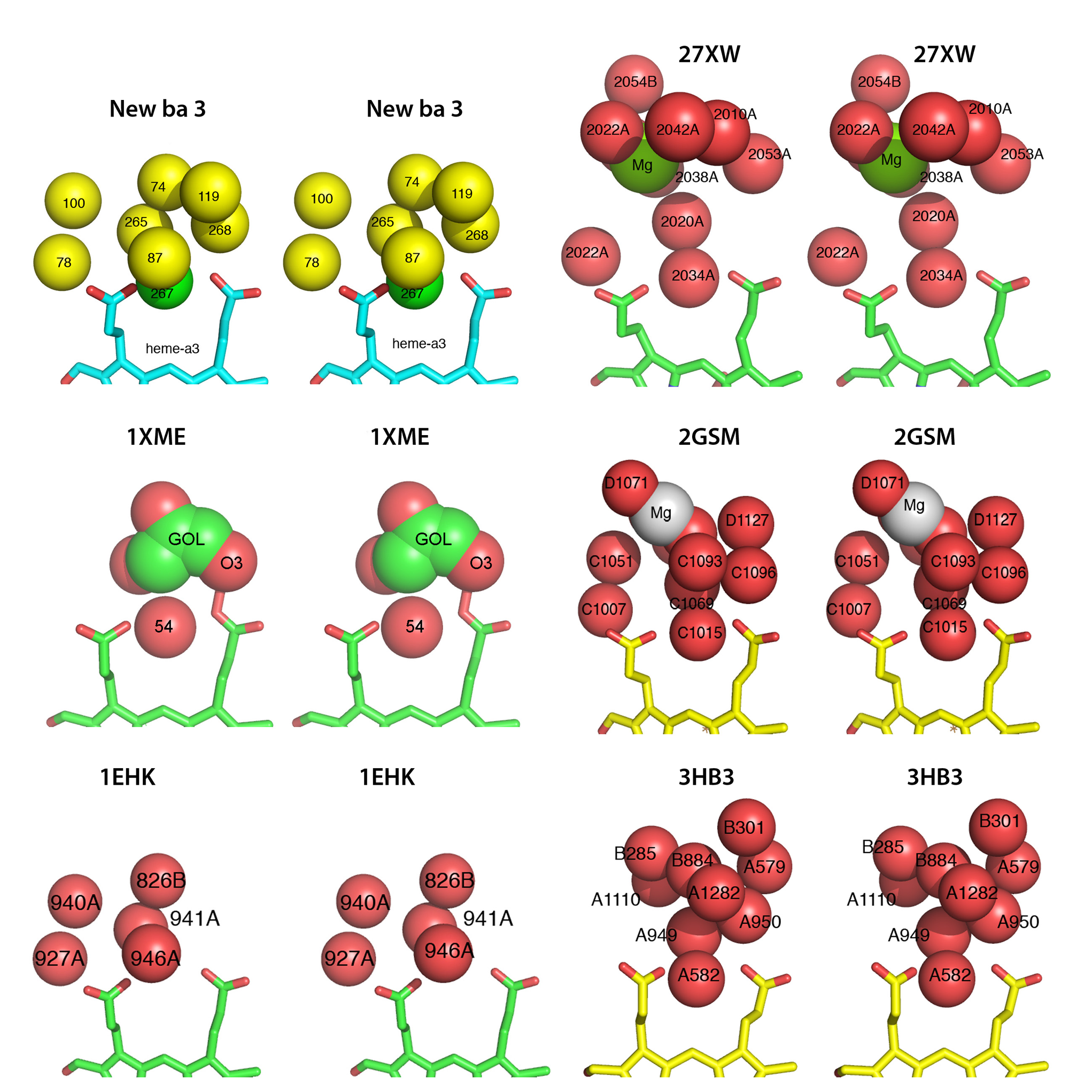

Supplement: Figure S6 — Stereo visual structural comparison of the inter-subunit water cluster in four different enzymes. The new ba3 structure (upper left); 1XME, ba3 with glycerol; 1EHK, ba3 original; 2ZXW, Bovine aa3; 2GSM, Rhodobacter sphaeroides aa3; and 3HB3, Paracoccus denitrificans aa3. (TIF) [file pone.0022348.s006.tif]

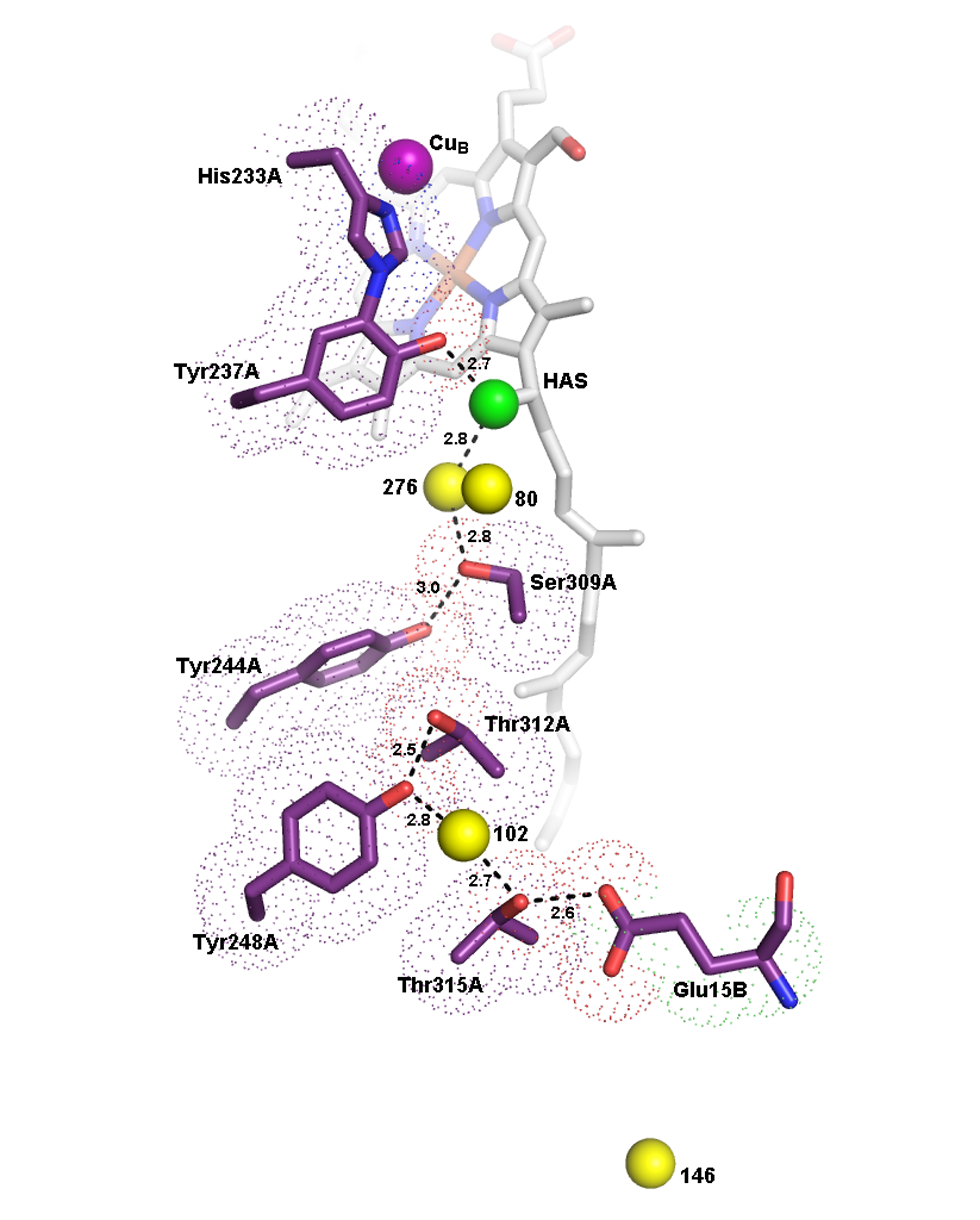

Supplement: Figure S7 — The proposed proton-uptake pathway (K path) in ba3 oxidase linking the cytosolic surface of the protein to the active center. Side chains of participating residues in subunits I and II (chains A, B) are purple, linking water molecules are yellow, and the secondary alcohol of the heme-a3 side chain is green. Oxygen atoms in the pathway are within hydrogen bonding distance of their nearest neighbors; the gap between Thr312A and Tyr244A (4.4 Å) is expected to be occupied by a not yet resolved water molecule. HOH80 is also within a hydrogen bonding distance to HOH276, Ser309A, and the secondary alcohol. HOH146 at the cytosolic surface of the protein can communicate with Glu15B, a strictly conserved residue, via a ∼9 Å long, loosely packed tube. Water molecules may also access Glu15B via Lys16B at the surface of the subunit II N-terminal transmembrane helix. (TIF) [file pone.0022348.s007.tif]

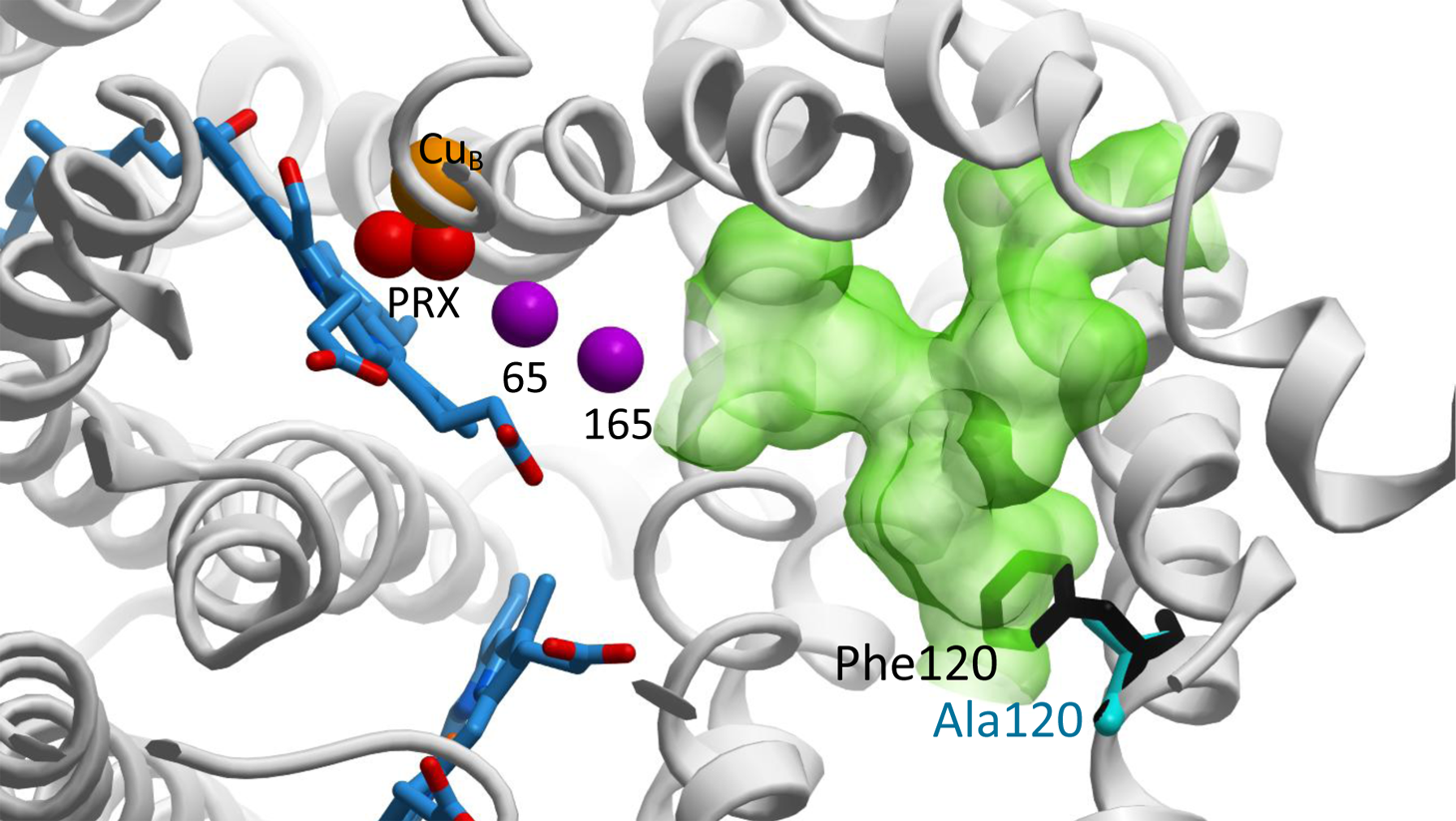

Supplement: Figure S8 — Comparison of the Y-shaped oxygen channel in the wild type ba3 and in the A120F mutant structures. The channel (green transparent surface) connects the active site of the enzyme with the protein- lipid interface. While in the WT structure both branches of the Y-shaped channel are opened to the surface of the protein (A120 shown in cyan sticks), the F120 side chain in the mutant protein (black sticks) completely blocks one of the openings. (TIF) [file pone.0022348.s008.tif]

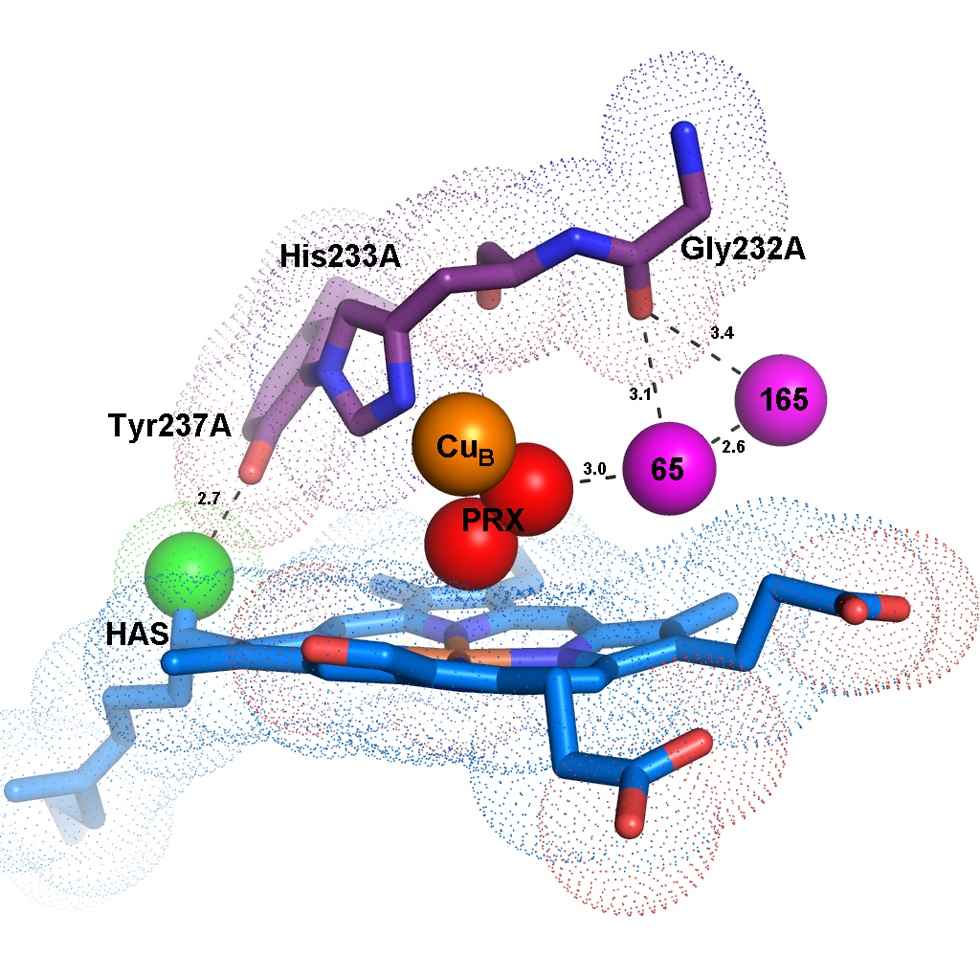

Supplement: Figure S9 — A close up of the active center. A chain of four oxygen atoms in the active center includes the peroxo dianion (red), coordinated to the Fe atom of heme-a3 (HAS) and the CuB atom (orange), and two water molecules, HOH65 and HOH165 (purple). HOH165 resides in the Xe1 site, i.e. an expected O2 binding site in the oxygen diffusion channel, nearest to the active center. HOH65 is hydrogen bonded to both the peroxo dianion and HOH165, and both water molecules can form hydrogen bonds with Gly232A in the oxygen diffusion channel. The hydrogen bond between the secondary alcohol of the heme-a3 side chain (green) and Tyr237A represents the terminus of the proton-uptake pathway (Figure S7). (TIF) [file pone.0022348.s009.tif]
