# Supplementary material for: High Resolution Structure of the ba3 Cytochrome c Oxidase from Thermus thermophilus in a Lipidic Environment
Source: PLoS One. 2011 Jul 21;6(7):e22348. doi: 10.1371/journal.pone.0022348 (PMC3141039; doi:10.1371/journal.pone.0022348)
Supplement: Table S1 — Assignment of lipids to clusters. (PDF) [file pone.0022348.s010.pdf]

Table S1. Assignment of lipids to clusters

**Cluster 1**	OLC3, OLC4, OLC15, OLC16, OLC19, OLC11*, OLC17*, OLC18*
**Cluster 2**	OLC5, OLC8, OLC9, OLC5*, OLC8*, OLC9*
**Cluster 3**	OLC6, OLC10, OLC6*, OLC10*
**Cluster 4**	OLC11, OLC12, OLC17, OLC18, OLC3*, OLC4*, OLC15*, OLC16*, OLC19*
**Cluster 5**	OLC1, OLC2, OLC7, OLC20
**Cluster 6**	OLC13, OLC14

* symmetry mates
