# Supplementary material for: High Resolution Structure of the ba3 Cytochrome c Oxidase from Thermus thermophilus in a Lipidic Environment
Source: PLoS One. 2011 Jul 21;6(7):e22348. doi: 10.1371/journal.pone.0022348 (PMC3141039; doi:10.1371/journal.pone.0022348)
Supplement: Table S2 — Comparison of ligand geometry to previous ba3 structures. (PDF) [file pone.0022348.s011.pdf]

Table S2. Comparison of ligand geometry to previous *ba*$_3$ structures

Distance (Å)	Current Structure	1XME	1EHK
NE of His384A to Fe of heme-a_3	2.2	2.5	3.3
Cu_B to Fe of heme-a_3	4.9	4.4	4.4
Bridging ion* to Fe of heme-a_3	2.39	2.4	2.3
Bridging ion* to Cu_B	2.25	2.1	2.3
CU1 of Cu_A to Fe of heme-*b*	19.2	19.2	19.0
CU1 of Cu_A to Fe of heme-a_3	21.8	21.7	21.8
CU1 of Cu_A to Cu_B	21.5	21.6	21.6
Fe of heme-*b* to Fe of heme-a_3	13.7	13.9	13.9
OH of Tyr237A to Fe of heme-a_3	5.7	5.8	5.6
Axial ligands His72A/NE2 and His386A/NE2 to heme-*b*	2.1	2.2	2.2
Separation of Cu_A atoms	2.6	2.5	2.6

***Bridging ion is a peroxide in the current structure and a water molecule in 1XME and 1EHK
